# Forensic Entomology in China and Its Challenges

**DOI:** 10.3390/insects12030230

**Published:** 2021-03-08

**Authors:** Yu Wang, Yinghui Wang, Man Wang, Wang Xu, Yanan Zhang, Jiangfeng Wang

**Affiliations:** Department of Forensic Medicine, Soochow University, Ganjiang East Road, Suzhou 215000, China; yuw@suda.edu.cn (Y.W.); ganyuchengge@163.com (Y.W.); wm15850051476@163.com (M.W.); 20194221067@stu.suda.edu.cn (W.X.); 20204221029@stu.suda.edu.cn (Y.Z.)

**Keywords:** forensic entomology, postmortem interval, development, succession, species identification

## Abstract

**Simple Summary:**

Forensic entomologists utilize sarcosaprophagous insect species to estimate the postmortem interval to aid death investigations. In this paper, we present the recent chronology of forensic entomology in China and illustrate how identification, development, and succession data are obtained and applied at the scale of such a large country. To overcome the difficulties and challenges forensic entomology faces in China, a number of countermeasures are provided.

**Abstract:**

While the earliest record of forensic entomology originated in China, related research did not start in China until the 1990s. In this paper, we review the recent research progress on the species identification, temperature-dependent development, faunal succession, and entomological toxicology of sarcosaprophagous insects as well as common applications of forensic entomology in China. Furthermore, the difficulties and challenges forensic entomologists face in China are analyzed and possible countermeasures are presented.

## 1. Origin and Development of Forensic Entomology in China

The book *Washing Away of Wrongs*, published during the Song dynasty, describes the earliest case report of forensic entomology [1]. No obvious progress was achieved in forensic entomology in China until the 1990s. In the 1990s, influenced by the boom of forensic entomology across Europe, North America, and Australia, Cui Hu and Hongzhang Zhou started exploratory research in southern and northern China, respectively [2]. With the publishing of *Forensic Entomology* by Hu [3], forensic entomology began to attract extensive attention in China. An array of studies focusing on species identification, temperature-dependent development, insect succession, and entomological toxicology have been conducted since then.

### 1.1. Species Identification

Forensic entomology represents the application of the study of insects (and other arthropods) to legal issues [4]. The most common use of entomological evidence in medicolegal investigations is the estimation of the time that has passed since death, which is referred to as the postmortem interval (PMI) [5]. The proper identification of forensically important species constitutes the first step and also the most crucial element in forensic entomology [6]. Species identification allows the application of the proper developmental data and insect succession patterns in an investigation [7]. If species determination is incorrect, the estimated PMI will also be incorrect [5]. Without the accurate identification of forensically important insect species, basic forensic entomology research is also not possible [2]. In China, entomologists conducted comprehensive and systematic work on the identification of sarcosaprophagous insects [8,9,10,11,12,13,14,15,16,17]. Most of their peer-reviewed findings have been absorbed into books. The *Key to the Common Flies of China (2nd Edition)* by Fan [18], published in 1992, contains 1547 common fly species in nine families and 250 genera. *Flies of China* by Xue and Zhao [19], published in 1996, contains 4209 fly species of 30 families and 660 genera. *Fauna Sinica Insecta Diptera: Calliphoridae* by Fan [20], published in 1997, contains five subfamilies, 48 genera, and 232 species of blow fly. These books lay a solid foundation for both research and application of forensic entomology. *Beetles Associated with Stored Products in China* by Zhang et al. [21], *Carrion Beetles of China (Coleoptera: Silphidae)* by Ji [22], and *Atlas of Chinese beetles: Staphylinidae* by Li [23] have laid the foundation for the identification of forensically important beetle species. However, research on the larval morphology of sarcosaprophagous beetles is still insufficient.

Chinese forensic entomologists have conducted many studies on the molecular identification of forensically important insect species and a wide range of mitochondrial and nuclear DNA markers has been identified [24,25,26,27,28,29,30,31]. Many taxa have been studied including Calliphoridae [26,27], Sarcophagidae [28,29,30], Muscidae [32], and several Coleoptera species [25]. Because the sole reliance on single DNA fragments for defining closely related species is perilous, multiple gene regions have been used for a more reliable diagnosis of forensically important flies [29]. Single-nucleotide polymorphisms (SNPs) have been used to aid the molecular identification of sarcosaprophagous flies [24]. The availability of complete mitochondrial genomes also offers an important basis for the identification and phylogenetic analysis of forensically important species [33,34]. Moreover, researchers found that cuticular hydrocarbon composition in the puparium can also be used for taxonomic differentiation of sarcosaprophagous flies [35].

In other countries, forensic entomologists have established useful keys for the morphological identification of forensically important insects [36,37,38,39]. These identification keys facilitate more detailed and species-specific knowledge of relevant species for forensic entomology experiments and real cases [36]. Although identification keys for forensically important insects were already established for taxonomic purposes in China [18,19,20,21,22,23], a more rigorous taxonomic foundation is still required for forensic purposes. In addition, molecular identification studies still need to sample taxa from different geographical regions and take better account of genetic intra- and interspecific variabilities.

### 1.2. Temperature-Dependent Development of Sarcosaprophagous Insects

Accurate and reliable temperature-dependent development data of insects form the basis for PMI estimation [40]. In China, the earliest developmental studies from the perspective of forensic entomology were launched in the 1990s. Ma et al. [41] and Wang [42] studied four and five common sarcosaprophagous fly species, respectively. For estimations of larval age, changes in body length and changes of morphological features (e.g., larval cuticle, cephalopharyngeal skeleton, and posterior spiracles) have been studied [42,43]. The results suggested that larval age can be estimated not only from the number of spiracular slits and changes of body length but also from the presence, thickness, color [42], and sclerotized area of the posterior peritreme [43]. In addition, the sclerotized area of the cephalopharyngeal skeleton is also a promising indicator for the estimation of larval age [43,44,45] (Table 1).

The concept of the minimum PMI (PMI_min_), as proposed by Amendt et al. [46], eliminates the uncertainty of the pre-colonization interval and provides a guideline for researchers to study the post-colonization interval of sarcosaprophagous insects. Consequently, they can establish more reliable development data for PMI_min_ estimation. To obtain more reliable and scientific results for the determination of the developmental patterns of sarcosaprophagous flies, recent studies of Chinese forensic entomologists have assessed wider temperature ranges and narrower sampling and observation intervals [47,48]. Moreover, forensic entomologists in China have also adopted more accurate temperature control devices and established additional developmental models for the estimation of PMI [49,50]. Investigated species include *Hemipyrellia ligurriens* (Wiedemann, 1830) [50], *Chrysomya megacephala* (Fabricius, 1794) [48], *Lucilia illustris* (Meigen, 1826) [47], *Parasarcophaga similis* (Meade, 1876) [51], *Boettcherisca peregrina* Robineau-Desvoidy, 1830 [52], *Calliphora grahami* (Aldrich, 1930) [53], *Musca domestica* Linnaeus, 1758 [54], *Chrysomya pinguis* (Walker, 1858) [55], *Muscina stabulans* (Fallén, 1817) [56], *Lucilia sericata* (Meigen, 1826) [57], *Chrysomya rufifacies* (Macquart, 1842) [58], *Hydrotaea spinigera* Stein, 1910 [59], and *Sarcophaga dux* Thomson, 1869 [49].

In flies, the intrapuparial period includes quiescent developmental stages that account for about half of the development time of the life cycle, which has important implications for accurate PMI_min_ estimation [60,61]. Chinese researchers have conducted several studies on morphological changes during the intrapuparial period and have divided this period into several events based on morphological and color changes [42,51,62,63,64,65,66,67,68]. The aim was to improve the accuracy of age estimation when using puparial samples. The investigated flies and other species include *C. megacephala* [63], *C. rufifacies* [68], Ca. *grahami* [42], *L. sericata* [42], *M. domestica* [42], *P. similis* [51], *L. cuprina* (Wiedemann, 1830) [62], *Megaselia spiracularis* [67], *Megaselia scalaris* (Loew, 1866) [66], *Dohrniphora cornuta* (Bigot, 1857) [64], and *Hermetia illucens* (Linnaeus, 1758) [65]. In particular, Wang et al. [69] conducted a study on the morphological changes of *L. illustris* during the intrapuparial period, which further identified detailed changes in various structures. The age of intrapuparial forms can not only be estimated from overall morphological changes but also be more accurately identified by the developmental processes of compound eyes, mouthparts, antennae, thorax, legs, wings, and the abdomen. These changes offer the potential to increase the accuracy of age estimation during the intrapuparial period [69].

In recent years, researchers worldwide have broadened the scope of their research in development biology. Particularly forensically important beetles have been gradually incorporated in their studies [70,71,72] as these beetles can be used to extend the estimation range of PMI when primary colonizers are no longer associated with the corpse or have emerged from puparia [73]. Chinese researchers also carried out temperature-dependent developmental studies on several forensically important beetles (e.g., *Creophilus maxillosus* (Linnaeus, 1758) [73], *Necrobia rufipes* (De Geer, 1755) [74], *Omosita colon* (Linnaeus, 1758) [75], and *Dermestes tessellatocollis* Motschulsky, 1860 [76]).

In addition to morphological observations, hemolymph soluble proteins [77], cuticular hydrocarbons [78], and differential gene expression [69,79,80,81] have also been applied to estimate the age of immature stages. Cuticular hydrocarbons in fly puparium were found to significantly change with weathering time, indicating their potential use for PMI_min_ estimation [82,83]. The use of gene expression level changes to estimate the age of stage has proven to be an effective approach to estimate the PMI_min_. The most prominent advantage of differential gene expression technology is that the obtained data represent quantitative results that can be subjected to error analysis, better conform to the Daubert test in forensic science, and are therefore more readily accepted by courts [84]. Notably, the combination of morphological observations with differential gene expression technology achieves higher estimation accuracy than either method alone [69,85]. Recently, researchers have studied the gene expression changes of *L. illustris* [69], *C. megacephala* [79], Ca. *grahami* [80], and *B. peregrina* [81] during the intrapuparial period and have further explored the gene expression changes of additional species at different stages. These studies lay a sound foundation for the molecular age estimation of sarcosaprophagous insects. Previous studies merely focused on establishing a connection between gene expression changes and development time. However, these studies commonly did not provide deep insight into the function of each differentially expressed gene and the molecular mechanism of phenotypic changes during growth and development [86]. In response to the rapid development of transcriptome sequencing technology and the increasing maturation of sequencing and data analysis techniques, research can now focus on developing transcriptome sequencing, gene screening, and bioinformatics analyses [86]. The generated data can be used to identify more optional, high-sensitivity genetic markers as candidates for molecular age estimation studies in forensic entomology [86]. These technologies can be expected to help establish more scientific and accurate methods for estimating the age of sarcosaprophagous insects.

The importance of a rigorous approach for the acquisition of reference development data for forensic applications has been highlighted by several researchers [46,87]. A standard approach that can be used to guide the development study is still missing in forensic entomology. Researchers in different regions or even within the same country often apply very different methods in their development studies. The divergence of these methodologies obstructs comparison of the results, thus limiting the exchange of data that may otherwise be helpful in forensic cases [88]. The studies of Bernhardt et al. [89] showed that not all tissues are similarly suitable for the gathering of sound growth data for sarcosaprophagous Diptera. Bernhardt et al. [89] suggested using minced pork as a non-human nutrition medium, since there are no developmental differences in this diet compared with human tissue. Bugelli et al. [90] found that the killing and storing methods of entomological samples can affect larval age estimation and suggested that the storing of maggots in 96% ethanol does not affect age estimation, or the specimen measurements should be done right after killing. The studies of Bernhardt et al. [89] and Bugelli et al. [90] provided important guidelines for the development of studies on forensic entomology. Further studies are needed to explore the effect of other factors with regard to the development of forensically important insects, such as fluctuating temperature and population competition between species, humidity, and photoperiod.

### 1.3. Faunal Succession of Insects

Different insect species visit and leave human remains in different orders and time sequences [97]. These regular insect activities are referred to as the faunal succession of insects and can be used to estimate the range of PMI [97]. Succession studies also provide information on the insects that occur in particular geographical regions or during particular seasons, which can be used to estimate the seasons and regions in which particular cases have taken place [2]. China has a vast territory with wide latitudinal coverage, complex geographical environments, and diverse climatic conditions. However, so far, only 20 insect succession studies have been published in 13 provinces (17 cities), accounting for about 1/3 of the total number of provincial-level administrative regions in China (Table 2).

Initially, most studies only addressed the species composition on remains in outdoor environments [98,99]. Later, researchers began to investigate the insect succession patterns under different environmental conditions [100,101,102]. The effects of indoor/outdoor environments [100], enclosed/unenclosed environments [103], exposure time [101], cadaver types [102], and toxicants [104] were explored with regard to body decomposition and insect succession. In particular, studies conducted in South China and the Yangtze River Delta region included not only the succession of adult insects but also the dynamic change processes of sarcosaprophagous insects on remains (such as egg laying, hatching, wandering, pupariation, eclosion, and disappearance) [102,105]. Insect succession matrixes of different seasons were obtained containing the residence times of different insects and their developmental stages [102,105].

Researchers used different types of models in insect succession studies [102,106,107]. Pig and rabbit carcasses were the most commonly used experimental remains, both of which were in nine studies. Three studies used animal tissues or organs to attract forensically important insects (Table 2). Human remains were used by four studies [98,99,102,108]. For instance, Zhou et al. [98] and Yang et al. [99] identified 38 species of sarcosaprophagous beetles and 14 species of sarcosaprophagous flies on human remains in Beijing. Chen [108] studied the body decomposition patterns and insect compositions on four human remains for four seasons under field conditions in Guizhou. Chen [108] applied the same method as used at the Anthropological Research Facility of the University of Tennessee, also known as the “Body Farm”. However, such a “body farm” does not exist in China yet. A study conducted in Shenzhen found that large pig carcasses, with similar weight to human corpses, also decomposed in a similar manner to human corpses; moreover, the species composition and succession pattern of insects were similar to that of human corpses. The carcasses of small pigs decomposed faster, attracted less insect species, and had a simpler succession pattern [102]. Rabbit carcasses could not fully reflect either the body decomposition or the changes of insects that occurred on a human corpse [102].

Research on the succession patterns of insects constitutes a number of the most fundamental tasks of forensic entomology [5,102], and the significance of such research is mainly embodied in the following three aspects: First, important basic data on species composition and succession patterns of sarcosaprophagous insects can be obtained via insect succession studies, which can be used to estimate the PMI [109,110]. Second, during succession studies, live insects with forensic importance can be collected and supplied as important colony sources for developmental research [53]. Third, researchers can accumulate precious practical experience, which cannot be otherwise obtained via laboratory studies. After weeks or months of body decomposition and insect succussion observations, forensic entomologists will have gained a clearer understanding of the decomposition processes of remains and will have acquired a basic time frame of the arrival orders of insects [2]. In addition, forensic entomologists will learn how to collect insect samples and where certain species of insects can be found [3]. With such knowledge and understanding, forensic entomologists can be more efficient and confident when dealing with real cases.

Forensic entomologists in different countries have conducted comprehensive work on the faunal succession of insects. Succession patterns have been identified under different types of environments (e.g., burying [111], vehicle environments [112], and dry environments [113]) and treatments (e.g., burning [114], hanging [115], and clothing [116]). Although these studies provided an important database, the difference between the insect fauna of China and other countries, as well as differences in other factors (e.g., climate and flora) justify the need for establishing patterns of insect succession for different regions of China [102]. Currently, insect succession data are not available for most parts of China. Most existing studies only concentrated on investigating species composition while failing to meet the requirements for establishing a succession matrix, which is required for PMI estimations. Considerable opportunity still remains for studying the faunal succession of insects in all parts of China.

### 1.4. Forensic Entomotoxicology in China

Chinese research on forensic entomotoxicology primarily focused on the effects of toxicants/drugs on the growth and development of insects. Tian [129] and Zhao et al. [130] found that morphine accelerated the development of flies and resulted in increased larval body length and weight [129], which caused a PMI deviation of up to 84 h [130]. Dai et al. [131] and Wang et al. [132] reported that diazepam accelerated fly development and shortened the larval development periods of *L. sericata* and *C. megacephala* by 55 and 60 h, respectively. Lv et al. [133] showed that ketamine inhibited the larval development rate of *C. megacephala* in a both dose-dependent and time-dependent manner. Zou et al. [134] showed that ketamine shortened the larval development period of *L. sericata*; the authors further showed that the development period of *L. sericata* larvae feeding on muscles and receiving ketamine at twice the lethal dose was 24 h shorter than that of control larvae. Liu et al. [135] reported that malathion inhibited the growth of larvae and puparia of *C. megacephala*, extended their developmental period by 36 h, and shortened their maximum body length by 1.1 mm. The study by Shi et al. [136] indicated that malathion and white arsenic affected the decomposition of carcasses by inhibiting insect colonization. Wang et al. [137] showed that the larval developmental time of Ca. *grahami* was significantly shorter on rabbit mince containing methamphetamine and further reported that methamphetamine can increase larval body length.

The effects of various toxicants/drugs on the growth and development of insects have also been studies by forensic entomologists in other countries. The results indicate that a number of toxicants/drugs, e.g., paracetamol [138], diazepam [139], cocaine [140], and codeine [141], can accelerate the development of forensically important insects. Other toxicants/drugs inhibited insect development, e.g., methamphetamine [142], malathion [143], alcohol [144], and amitriptyline [145]. Desmethyldiazepam [146], nandrolone [147], and gentamicin [148] did not significantly change the growth and development of sarcosaprophagous insects. The results obtained by Chinese researchers were consistent with the above results, and the same effects were found on insect development in the same toxicants/drugs. Forensic entomotoxicology was once extensively studied as a branch of forensic entomology from the end of the 20th century to the beginning of the 21st century; however, studies in this field have been declining over recent years. One of the main reasons restricting the development of forensic entomotoxicology is that although qualitative detection has been achieved, quantitative analyses still remain problematic [149]. Further studies are still required to overcome existing bottlenecks.

## 2. Applications of Forensic Entomology in China

The *Regulations on the Classification of the Practice of Forensic Judicial Appraisal*, released by the Chinese Ministry of Justice on 9 May 2020, stipulates that insects can serve as evidence for PMI estimation. This regulation establishes the legal status of forensic entomology. However, Chinese judicial or police departments have no full-time forensic entomology positions. Forensic entomologists are mainly researchers working at institutions of higher education or employees of research institutions, who may participate in cases as expert witnesses similar to other countries. When forensic entomologists are asked to provide expert opinions that may ultimately decide how cases are adjudicated, they will be invited to attend field investigations. In most circumstances, the police will only mail specimens or send photos of related insects to forensic entomologist, combined with brief details of the case. The resulting PMI estimated is then only used to narrow the scope of the police investigation. China has not yet established specialized academic institutions for forensic entomology research, and researchers are most commonly members of the Entomological Society of China or the Forensic Medicine Association of China. A few universities are offering forensic entomology education to M.Sc. and Ph.D. students, while undergraduate education is still not available. Because of this lack of full-time forensic entomology posts, most interested postgraduates have to consider career changes. So far, fewer than a dozen researchers are qualified to present judicial expertise on forensic entomology in the entirety of China.

Currently, forensic entomology is only applied in a few regions of China. The identification of insect species is largely based on morphological identification keys provided by entomologists [150,151]. Peer-reviewed development and succession data are used to estimate the PMI [54,150,151]. Plasticity of developmental rates has been reported between different colonies of the same species [152,153]; therefore, in applications with multiple literature reports on the development of one species, the development data obtained from the same or a nearby geographical region will be prioritized [154]. Succession data from the same or similar regions and months will be used to estimate the PMI [155].

More than 23 case reports of the application of forensic entomology in China have been published [54,58,150,151,154,155,156,157,158,159,160,161,162,163,164]. The most widespread application of forensic entomology is the provision of PMI clues for criminal investigators [58,150,151,154,155,156,157,158,159,160,163,164]. Forensic entomology also played an important role in an insurance compensation case [54]. There were 14 reported cases in the outdoor environment [58,150,151,154,155,156,157,160,161,162,163,164] and nine cases occurred indoors [54,151,155,158,159]. The development duration and larval body length of flies were the most commonly used indicators for the PMI estimation, both of which were utilized in 10 cases [54,58,150,151,154,155,156,159,161,162]. Three cases utilized the thermal summation constant of flies to estimate the PMI [158,160]. The development and succession patterns of beetles were only utilized for PMI estimation in one case [155]. Other cases used the biological characteristics of forensically important blow flies to estimate the PMI. On the basis of the seasonal distribution characteristics of *C. pinguis* and Ca. *grahami*, Li et al. [162] inferred the initial occurrence time of two fly species (from the end of February to the beginning of March) as the most likely time frame of PMI of the deceased. The actual result confirmed this inference. In a case reported by Hu et al. [164], a cadaver, found in a suitcase, contained *H. illucens*, *Me. scalaris*, and *Fannia canicularis*, while blow flies were absent. Analysis indicated that the deceased likely began to decompose during winter, and that, as the weather warmed, the cadaver was already highly decomposed and no longer attracted calliphorid species (as these prefer fresh remains). On this basis, it could be inferred that the PMI of the corpse started at the beginning of winter, which was later confirmed by investigation results.

## 3. Challenges for Forensic Entomology in China and Proposed Countermeasures

Forensic entomology faces many challenges in China. First, new technologies, such as video surveillance technology, DNA technology, and big data technology, have developed rapidly over the past few years [165]. These technologies contribute to the increase of police detection rates, and many of them can help to estimate the PMI [2,165]. For example, if the decedent was identified by the technology of DNA, his/her debit and credit card expenditures and telephone, accommodation, and traffic records can provide a clue for PMI. Impacted by various newly emerging case-solving technologies, the demand of criminal investigators for forensic entomology has decreased [2]. Second, forensic entomologist usually cannot provide a precise PMI result as forensic entomology is limited and can only provide a PMI_min_ or a PMI range [166]. These usually deviate by days or even months from the real PMI [151], thus making it difficult for criminal investigators to fully accept the value of forensic entomology. Third, a number of criminal investigators do not have the basic knowledge of forensic entomology and might not follow the recommended standards and practices in forensic entomology. This potentially limits the accuracy of the obtained PMI estimations. For example, the insect evidence provided may not include the first arriving species or the oldest immature insects. Without reliable and sufficient insect evidence, forensic entomologists cannot provide an accurate result. Therefore, it would be advisable if forensic entomologists could be present at the scene for specimen collection, as no one else can fully understand the importance of each piece of insect evidence.

However, forensic entomologists cannot attend every case. Consequently, education and training need to be strengthened to enhance the ability and understanding of the criminal investigators who are frequently involved in cases that would benefit from forensic entomology. Most investigators do not consider using forensic entomology when encountering death cases. The reason most often is that they are not familiar with forensic entomology or think that the application of forensic entomology is too complex. Therefore, an urgent need for education and training exists to help these investigators to understand the theory of forensic entomology, e.g., why temperature data is so important, under which circumstances forensic entomology can be utilized, and how entomological evidence is collected correctly. To achieve this, Soochow University has offered a course of 36 credit hours to undergraduate students with specialization in forensic sciences. The purpose of this course is not for students to become competent forensic entomologists but to enable them to correctly collect insect evidence or at least to induce their proactivity for asking for forensic entomologists in their future work. Forensic entomology researchers should also be trained. Some researchers cannot correctly identify the species of sarcosaprophagous insects, especially when these are immature. Some researchers have never performed basic experimental studies and/or lack the required expertise to analyze the entomological evidence associated with a case. Hence, more comprehensive training should be offered to help them overcome application barriers.

In the application, forensic entomologists should try their best to provide a scientifically informed PMI estimate. Although the PMI estimate may not absolutely coincide with the actual PMI, it is better if the real PMI falls within the scope of the estimated PMI. To improve the accuracy of PMI estimation, the following conditions must be met: (1) all entomological evidence must be collected systematically and comprehensively; (2) case information and environmental information must be understood systematically and comprehensively; furthermore, weather data must be retrieved, and accurate environmental data must be obtained through field measurements and corrections; (3) the species and stages of all insects present at the scene must be correctly identified; (4) adequate laboratory work must be conducted and accurate reference data on insect development must be obtained; (5) investigations of insect succession must be conducted in similar regions; (6) various types of cases need to be studied to accumulate experience; (7) all cases should be pre-appraised by forensic entomologists. If the insect evidence is not provided correctly and appropriately by criminal investigators, forensic entomologists should request an additional collection. Otherwise, PMI estimation should be refused. Cases that exceed the scope of forensic entomology should also be rejected to avoid mistakes.

## 4. Conclusions

Over nearly 30 years of exploration and development, forensic entomology has begun to play an increasingly important role in forensic practices; however, it is still far from the ultimate goal of achieving universal application in China. Faced with the challenge of various newly emerging case-solving technologies, if forensic entomology wants to further develop and progress, basic research must be strengthened. Further studies should be conducted to establish more accurate development data, based not only on morphological methods but also on techniques of differential gene expression, biochemical properties, and artificial intelligence. Moreover, basic data of succession patterns of insects should be obtained in more environments and regions, to further probe into the mechanisms of colonization of corpses by insects. Both training and education of criminal investigators should be strengthened to promote the application of forensic entomology.

## Figures and Tables

**Table 1:** Summary of studies that reported the development of sarcosaprophagous insects in China.

Order	Species	Citation	City (Province)	Temperatures (°C)	Indicators
Diptera	*Boettcherisca peregrina* Robineau-Desvoidy, 1830	Wang et al. [42]	Hangzhou (Zhejiang)	16, 20, 24, 28, 32	Dd, Lbl, T_0_, K, Imc, Lmc
		Wang et al. [91]	Guangzhou (Guangdong)	15, 20, 25, 30, 35	Dd, Lbl, Lbw, Lbwi, Wp
		Wang et al. [52]	Suzhou (Jiangsu)	16, 19, 22, 25, 28, 31, 34	Dd, Ihen, Lbl, Ilen, T_0_, K
		Shang et al. [85]	Changsha (Hunan)	15, 25, 35	Ige
	*Calliphora grahami* (Aldrich, 1930)	Ma et al. [41]	Hangzhou (Zhejiang)	12,15, 18, 21, 24, 27, 30	Dd
		Wang et al. [42]	Hangzhou (Zhejiang)	12, 16, 20, 24, 28	Dd, Lbl, T_0_, K, Imc, Lmc
		Zhao et al. [92]	Shijiazhuang (Hebei)	16, 20, 24, 28, 32	Lmc
		Wang et al. [53]	Suzhou (Jiangsu)	16, 19, 22, 25, 28, 31, 34	Dd, Ihen, Lbl, Ilen, T_0_, K
		Liu et al. [84]	Changsha (Hunan)	15, 22, 27	Ige
		Chen et al. [93]	Changsha (Hunan)	Constant vs. fluctuating temperature (8 vs. 6–12; 12 vs. 10–16; 16 vs. 14–20)	Dd, Ihen, Lbl, Ilen, T_0_, K
	*Chrysomya megacephala* (Fabricius, 1794)	Ma et al. [41]	Hangzhou (Zhejiang)	18, 21, 24, 27, 30, 33	Dd
		Wang et al. [42]	Hangzhou (Zhejiang)	16, 20, 24, 28, 32	Dd, Lbl, T_0_, K, Imc, Lmc
		Zhao et al. [92]	Shijiazhuang (Hebei)	16, 20, 24, 28, 32	Lmc
		Yang et al. [48]	Chongqing (municipality)	16, 19, 22, 25, 28, 31, 34	Dd, Ihen, Lbl, Ilen, T_0_, K
		Zhang et al. [63]	Suzhou (Jiangsu)	16, 19, 22, 25, 28, 31, 34	Dd, Ihen, Lbl, Ilen, T_0_, K, Imc
		Wang et al. [83]	Suzhou (Jiangsu)	22.5, 27.5, 32.5	Ige
	*Chrysomya nigripes* Aubertin, 1932	Li et al. [94]	Guangzhou (Guangdong)	20, 24, 28, 32	Dd, Lbl, Lbw
	*Chrysomya pinguis* (Walker, 1858)	Zhang et al. [55]	Suzhou (Jiangsu)	16, 19, 22, 25, 28, 31, 34	Dd, Ihen, Lbl, Ilen, T_0_, K
	*Chrysomya rufifacies * (Macquart, 1842)	Ma et al. [68]	Guangzhou (Guangdong)	20, 24, 28, 32	Imc
		Hu et al. [58]	Suzhou (Jiangsu)	16, 19, 22, 25, 28, 31, 34	Dd, Ihen, Lbl, Ilen, T_0_, K
	*Dohrniphora cornuta * (Bigot, 1857)	Feng et al. [64]	Shenyang (Liaoning)	15, 18, 21, 24, 27, 30, 33, 36	Imc
	*Hemipyrellia ligurriens* (Wiedemann, 1830)	Yang et al. [50]	Chongqing (municipality)	16, 19, 22, 25, 28, 31, 34	Dd, Ihen, Lbl, Ilen, T_0_, K
	*Hermetia illucens* Linnaeus, 1758	Li et al. [65]	Guangzhou (Guangdong)	20, 24, 28, 32	Dd, Imc
	*Hydrotaea spinigera* Stein, 1910	Wang et al. [59]	Suzhou (Jiangsu)	16, 19, 22, 25, 28, 31, 34	Dd, Ihen, Lbl, Ilen, T_0_, K
	*Lucilia cuprina * (Wiedemann, 1830)	Wang et al. [62]	Shijiazhuang (Hebei)	16, 20, 24, 28, 32	Imc
	*Lucilia illustris* (Meigen, 1826)	Wang et al. [47]	Suzhou (Jiangsu)	15, 17.5, 20, 22.5, 25, 27.5, 30, 32.5, 35	Dd, Ihen, Lbl, Ilen, T_0_, K
		Wang et al. [69]	Suzhou (Jiangsu)	20, 25, 30	Imc, Ige
	*Lucilia sericata * (Meigen, 1826)	Ma et al. [41]	Hangzhou (Zhejiang)	18, 21, 24, 27, 30, 33	Dd
		Wang et al. [42]	Hangzhou (Zhejiang)	16, 20, 24, 28, 32	Dd, Lbl, T_0_, K, Imc, Lmc
		Li [43]	Shijiazhuang (Hebei)	16, 20, 24, 28, 32	Lmc
		Wang et al. [57]	Suzhou (Jiangsu)	16, 19, 22, 25, 28, 31, 34	Dd, Ihen, Lbl, Ilen, T_0_, K
	*Megaselia scalaris * (Loew, 1866)	Feng and Liu [66]	Shenyang (Liaoning)	18, 21, 24, 27, 30, 33, 36	Imc
	*Megaselia spiracularis* Schmitz, 1938	Feng and Liu [67]	Shenyang (Liaoning)	21, 24, 27, 30, 33, 36	Imc
		Wang et al. [95]	Suzhou (Jiangsu)	16, 19, 22, 25, 28, 31, 34	Dd, Ihen, Lbl, Ilen, T_0_, K
	*Musca domestica* Linnaeus, 1758	Wang et al. [42]	Hangzhou (Zhejiang)	16, 20, 24, 28	Dd, Lbl, T_0_, K, Imc, Lmc
		Wang et al. [54]	Suzhou (Jiangsu)	16, 19, 22, 25, 28, 31, 34	Dd, Ihen, Lbl, Ilen, T_0_, K
	*Muscina stabulans* (Fallén, 1817)	Wang et al. [56]	Suzhou (Jiangsu)	16, 19, 22, 25, 28, 31, 34	Dd, Ihen, Lbl, Ilen, T_0_, K
	*Parasarcophaga crassipalpis* (Macquart, 1938)	Ma et al. [41]	Hangzhou (Zhejiang)	18, 21, 24, 27, 30, 33	Dd
		Wang et al. [44]	Shijiazhuang (Hebei)	16, 20, 24, 28, 32	Lmc
	*Parasarcophaga similis* (Meade, 1876)	Yang et al. [51]	Suzhou (Jiangsu)	16, 19, 22, 25, 28, 31, 34	Dd, Ihen, Lbl, Ilen, T_0_, K, Imc
	*Sarcophaga dux* Thomson, 1869	Zhang et al. [49]	Changsha (Hunan)	16, 19, 22, 25, 28, 31, 34	Dd, Ihen, Lbl, Ilen, T_0_, K, Ige
Coleoptera	*Creophilus maxillosus * (Linnaeus, 1758)	Wang et al. [73]	Suzhou (Jiangsu)	17.5, 20, 22.5, 25, 27.5, 30, 32.5	Dd, Ihen, Lbl, Ilen, T_0_, K
	*Necrobia rufipes* (De Geer, 1755)	Hu et al. [74]	Suzhou (Jiangsu)	22, 25, 28, 31, 34, 36	Dd, Ihen, Lbl, Ilen, T_0_, K, LD
	*Omosita colon* (Linnaeus, 1758)	Wang et al. [75]	Suzhou (Jiangsu)	16, 19, 22, 25, 28, 31	Dd, Ihen, T_0_, K
Hymenoptera	*Nasonia vitripennis* (Walker, 1836)	Zhang et al. [96]	Suzhou (Jiangsu)	16, 19, 22, 25, 28, 31, 34	Dd, Ihen, Lbl, T_0_, K

Abbreviations: Dd: developmental duration; Ihen: isomorphen diagram; Lbl: larval body length; Ilen: isomegalen diagram; Lbw: larval body weight; Lbwi: larval body width; Wp: weight of puparia; T_0_: developmental threshold temperature; K: thermal summation constant; Imc: intrapuparial morphological changes; Ige: intrapuparial gene expression; LD: larval instar determination; Lmc: larval morphological changes.

**Table 2:** Summary of studies reporting body decomposition and insect succession in China.

Citation	City (Province)	Experimental Model (Numbers of Remains)	Season/Month	Insect Species/Other Information
Zhou et al. [98]	Beijing (capital)	Human corpse (NS)	March to August	Coleoptera: 38 species
Yang et al. [99]	Beijing (capital)	Human viscera (NS)	March to November	Diptera: 14 species
Ma et al. [117]	Hangzhou (Zhejiang)	Pork meat (NS)	All seasons	Diptera: 12 species Coleoptera: 16 species Hymenoptera: 2 species
Li et al. [118]	Harbin (Heilongjiang)	Pork meat (NS)	Spring, summer, and autumn	Diptera: 8 species Coleoptera: 19 species
Chen et al. [119]	Twelve sites (Guizhou)	Pork lung (NS)	All seasons	Diptera: 27 species
Wang et al. [120]	Chengdu (Sichuan)	Rabbit (28)	1st year: May to November 2nd year: March to September	Diptera: 5 species
Chen [121]	Zhongshan (Guangdong)	Pig (4)	Autumn and winter	Total: 38 species
Chang et al. [106]	Hohhot (Inner Mongolia)	Rabbit (25), Dog (1)	July to October	Diptera: 10 species
Wang et al. [105]	Pearl River Delta (Guangdong)	Pig (18)	All seasons	Diptera: 17 species Coleoptera: 16 species Other: 9 species
Wu et al. [122]	Guangzhou (Guangdong)	Rabbit (NS)	Spring and summer	Diptera: 10 species Coleoptera: 7 species
Dong et al. [123]	Sanmenxia (Henan)	Rabbit (5)	July to October	Three families, 13 species
Chen et al. [108]	Guiyang (Guizhou)	Human corpse (4)	All seasons	Diptera: 11 species
Nie et al. [124]	Xi’an (Shanxi)	Rabbit (4)	Spring	Diptera: 10 species Coleoptera: 4 species Other: 2 species
Shi et al. [104]	Guangzhou (Guangdong)	Rabbit (4)	Summer	Effects of malathion on the insect succession
Jiang et al. [125]	Yongzhou (Hunan)	Rabbit (9)	July to September	Total: 26 species
Yin et al. [100]	Shenzhen (Guangdong)	Pig (4)	Summer	Indoor: 14 species Outdoor: 18 species
Jiang et al. [126]	Qingdao (Shandong)	Pig (12)	All seasons	Diptera: 23 species
Lv [127]	Chongqing (municipality)	Pig (11)	All seasons	Insecta: 94 species
Li et al. [103]	Guangzhou (Guangdong)	Pig (2)	Summer	Comparative study of carcasses between enclosed and open-air conditions
Yang [128]	Suzhou (Jiangsu)	Pig (22)	Summer and autumn	Diptera: 16 species Coleoptera: 12 species Other: 5 species
Wu et al. [107]	Xinxiang (Henan)	Rabbit (5), rat (6)	July to August	Diptera: 7 species
Wang et al. [101]	Guangzhou (Guangdong)	Pig (6)	Summer	Insect succession on pig carcasses using different exposure times
Wang et al. [102]	Shenzhen (Guangdong)	Human corpse (1), large pig (2), small pig (2), rabbit (2)	August to December	Total: 42 species; insect assemblages are more complex on larger carcasses, following the order of human = large pig > small pig > rabbit

NS: not specified.
